# Anti-stress effects of Fameyes in in vitro and in vivo models of stresses

**DOI:** 10.1186/s42826-022-00149-w

**Published:** 2022-12-06

**Authors:** Junkee Hong, Tae-Kyeong Lee, In Hye Kim, Seungah Lee, Byung-Ju Jeon, Jiwon Lee, Moo-Ho Won, Sungsu Kim

**Affiliations:** 1 Precision Medicine R&D Center, Famenity Co., Ltd., Uiwang, Gyeonggi 16006 Republic of Korea; 2 Department of Food Science and Nutrition, Hallym University, Chuncheon, Gangwon 24252 Republic of Korea; 3 R&D Center, Naturesense INC., Ltd., Uiwang, Gyeonggi 16006 Republic of Korea; 4 Department of Neurobiology, School of Medicine, Kangwon National University, Chuncheon, Gangwon 24341 Republic of Korea

**Keywords:** Immobility, ACTH, Antioxidant, Cortisol, Fatigue, Relaxation, Serotonin

## Abstract

**Background:**

Fameyes (a mixture of *Clematis mandshurica* Rupr. extract (CMRE) and *Erigeron annuus* (L.) Pers. extract (EAPE)) containing scutellarin and chlorogenic acid as major components has been reported to relieve mental stress in human subjects, which is reflected in improved scores in psychometric tests measuring levels of depression, anxiety, well-being, and mental fitness. The aim of this study was to examine the anti-stress activity of Fameyes and to investigate the mechanisms of the anti-stress activity using in vitro and in vivo models of stresses.

**Results:**

First, we tested the effect of Fameyes on corticosterone-induced cytotoxicity in SH-SY5Y cells (human neurofibroma cell lines). Corticosterone induced apoptosis and decreased cell viability and mitochondrial membrane potential, but treatment with Fameyes inhibited these cytotoxic effects in a dose-dependent manner. However, CMRE and EAPE (components of Fameyes) did not inhibit the cytotoxic effect of corticosterone individually. Next, we tested the effects of Fameyes on rats that were exposed to different kinds of stresses for four weeks. When the stressed rats were treated with Fameyes, their immobility time in forced swim and tail suspension tests decreased. A reduction was also observed in the serum levels of adrenocorticotropic hormone (ACTH) and corticosterone. Furthermore, upon oral administration of Fameyes, serum serotonin levels increased. These in vitro and in vivo results support the anti-stress effects of Fameyes.

**Conclusions:**

In vitro experiments showed anti-stress effects of Fameyes in cell viability, apoptosis, and mitochondrial membrane potential. In addition, in vivo experiments using rats showed anti-stress effects of Fameyes in blood and tissue levels of ACTH, corticosterone, and serotonin, as well as the immobility time in the forced swim and tail suspension tests. However, we did not specifically investigate which ingredient or ingredients showed anti-stress effects, although we reported that Fameyes contained chlorogenic acid and scutellarin major ingredients.

## Background

It is widely accepted that stress causes several physical and mental disorders by disrupting the homeostasis of the body. Since stress has increased with the rapid industrialization and high economic growth, people in developed countries experience more stress; thus, it has become a serious health problem that must be overcome [1, 2]. Unfortunately, there is no standard therapy for managing or coping with stress. Although some antidepressants are available, they cause adverse side effects [3].

The mechanism through which stress affects the body is still unclear. However, hormones secreted from the hypothalamic-pituitary–adrenal axis have been suggested to play important roles in stress-related adverse effects. Physical and mental stress stimulates the anterior pituitary gland (or adenohypophysis) to secrete adrenocorticotropic hormone (ACTH), which in turn stimulates the adrenal cortex to secrete corticosteroids including corticosterone and cortisol. Simultaneously, stress suppresses the secretion of serotonin from the neurons in the raphe nucleus located along the midline of the brainstem [4, 5].

Corticosterone and cortisol shift the metabolic balance from an anabolic state to a catabolic state involving sodium retention and immunosuppression. The catabolic state is closely related to muscle depletion, osteoporosis, hyperlipidemia, obesity, and diabetes [6, 7]. Sodium retention is an important factor that can lead to hypertension, and immunosuppression increases the risk of various infections [6, 7]. Serotonin is known to mediate the feeling of happiness, and the lower level of serotonin can cause a shift from a calm, happy, and energized state to that of tension, anxiety, depression, and fatigue [8, 9].

Fameyes is a mixture of leaf extracts of *Clematis mandshurica* Rupr. (CMR) and *Erigeron annuus* (L.) Pers. (EAP). CMR called buttercup has traditionally been used as a food additive and remedy for various diseases [10]. Recently, the ethanol extract of CMR was reported to inhibit the release of inflammatory mediators from lipopolysaccharide-treated macrophages and suppressed inflammatory reactions in delayed-type hypersensitivity in mice [10]. EAP called daisy fleabane is traditionally known for its antipyretic, detoxifying, anti-inflammatory, and blood-sugar-lowering properties [11, 12]. Fameyes has been reported to protect against ischemia–reperfusion injury in rodent brains, suggesting its ROS-scavenging property [13, 14]. In addition to suppressing oxidative stress in brain tissues, Fameyes was observed to relieve mental stress in human subjects [15]. Furthermore, it was recently reported as a reactive oxygen species (ROS) scavenger [11]. In this study, oral administration of YES‑10® (200 mg) for four weeks substantially improved the test scores of the patients on the Beck Depression Inventory, Beck Anxiety Inventory, Psychosocial General Well-Being Index, and Mental Fitness Scale, which are psychometric tests to measure levels of depression, anxiety, well-being, and mental fitness, respectively [15].

The above evidence for the stress-relieving property of YES‑10® was obtained on the basis of the subjective responses of the participants. Therefore, to confirm the anti-stress activity of YES‑10®, objective evidence should be gained from biochemical and pathological studies. In the present study, the effects of YES‑10® were tested on corticosterone-treated neuroblastoma cells and physically stressed rats. The in vitro experiments were performed to study the effects of Fameyes on cell viability, apoptosis, and mitochondrial membrane potential (MMP). Meanwhile, in the in vivo experiments, the effects of Fameyes on stressed rats were assessed on the basis of blood and tissue levels of ACTH, corticosterone, and serotonin, as well as the immobility time in the forced swim and tail suspension tests.

## Results

### Protective effects of Fameyes against corticosterone-induced cytotoxicity

**Fig. 1:**
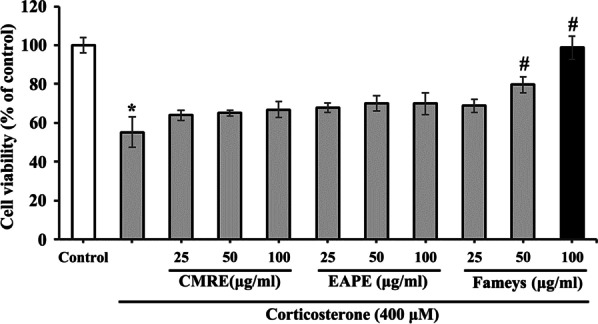
Effects of CMRE, EAPE and Fameyes on the viability of the corticosterone-treated SH-SY5Y cells. Corticosterone (400 μM) reduces cell viability by 50%. However, 50 and 100 μg/mL Fameyes restores cell viability to 79.7% and 98.7%, respectively. Each bar indicates the means ± S.E.M. (*n* = 5, respectively). *P < 0.05; compared with control group. ^#^*P* < 0.05; compared with corticosterone-treated group

**Fig. 2:**
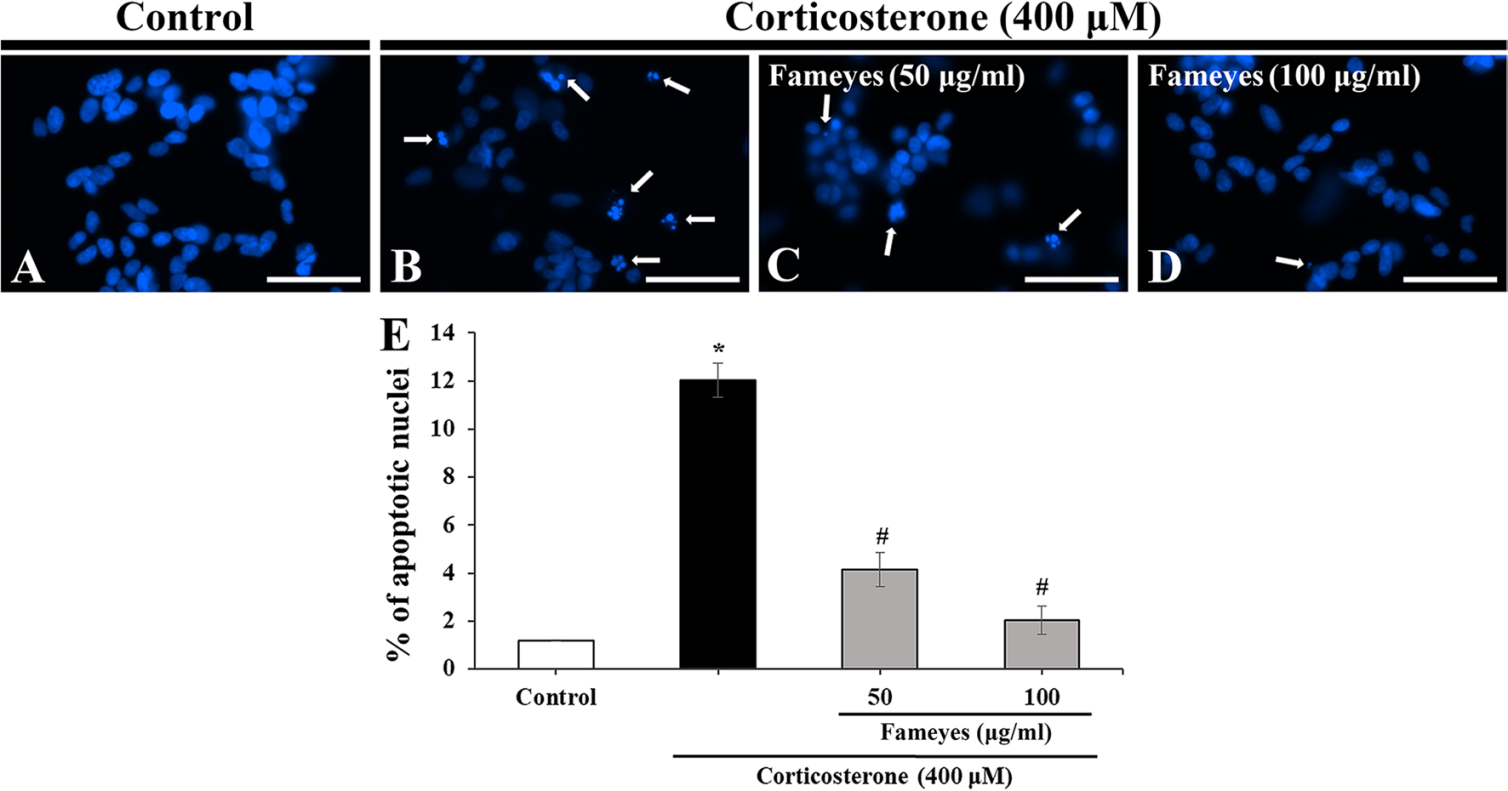
Effects of Fameyes on apoptosis of corticosterone-treated SH-SY5Y cells in the Control (**A**), 400 μM corticosterone (**B**), 400 μM corticosterone + 50 μg/ml YES (**C**) and 400 μM corticosterone + 100 μg/ml YES (**D**) groups. Corticosterone induces apoptosis in SH-SY5Y cells, but, at 100 μg/mL Fameyes, apoptosis is almost completely prevented. Arrows indicate the apoptotic cells. (**E**) percentage of apoptotic cells of all the groups. Scale bar = 50 μm

**Fig. 3:**
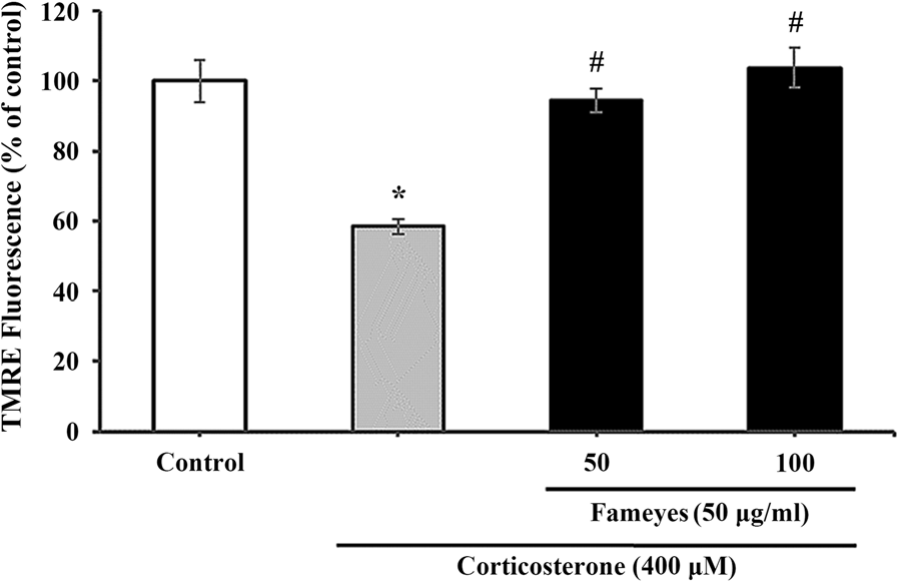
Effects of Fameyes on MMP of corticosterone-treated SH-SY5Y cells. MMP is reduced to 58.4% by corticosterone; the recovery is almost 100% at 100 μg/mL Fameyes. Each bar indicates the means ± S.E.M (*n* = 3, resspectively). *P < 0.05; compared with control group. ^#^*P* < 0.05; compared with corticosterone-treated group

As shown in Fig. 1, the effects of Fameyes on the corticosterone-induced cytotoxicity of SH-SY5Y cells (human neuroblastoma cell lines) were evaluated in terms of three aspects: cell viability, apoptosis, and MMP. Compared to cell viability of the control group, treatment with corticosterone (400 μM) reduced cell viability by 50%. However, 50 and 100 μg/mL Fameyes restored cell viability to 79.7% and 98.7%, respectively. A lower concentration of Fameyes (25 μg/mL) did not exhibit any considerable effect. These results indicate that cytotoxicity induced by corticosterone was reduced by Fameyes treatment. Fameyes is a mixture of the extract of the leaves of *Clematis mandshurica* RUPR. (CMRE) and the extract of leaves of *Erigeron annuus* (L.) PERS. (EAPE); thus, the effects of its individual component were compared to those of Fameyes. Treatment with only CMRE or EAPE did not show any statistically significant results (Fig. 1). These results show that CMRE and EAPE alone were not remarkably effective in protecting cells. Therefore, in the apoptosis (Fig. 2) and MMP (Fig. 3) experiments, only the effects of Fameyes were tested. When morphological changes in the nuclei of the cells caused by corticosterone treatment were observed, apoptotic bodies were observed (Fig. 2B). In this group, the percentage of apoptotic cells was 12.0% (Fig. 2E). On the other hand, the SH-SY5Y cells treated with corticosterone and Fameyes showed reduction of apoptotic bodies dose-dependently, showing that the percentages of the apoptotic cells were 4.1% and 2.1%, respectively (Fig. 2C–E). In other words, treatment with Fameyes significantly decreased apoptotic cell death by corticosterone cytotoxicity (Fig. 2E). Similar results were obtained in the MMP study. MMP was reduced to 58.4% by corticosterone and recovered by Fameyes in a dose-dependent manner. The recovery was almost 100% at 100 μg/mL (Fig. 3). The results obtained from these cellular studies indicate that Fameyes protects against the cytotoxic actions of corticosterone. Interestingly, this action was exhibited only by Fameyes and not by its components, CMRE and EAPE, individually.

### Effects of Fameyes on immobility time of stressed rats

**Fig. 4:**
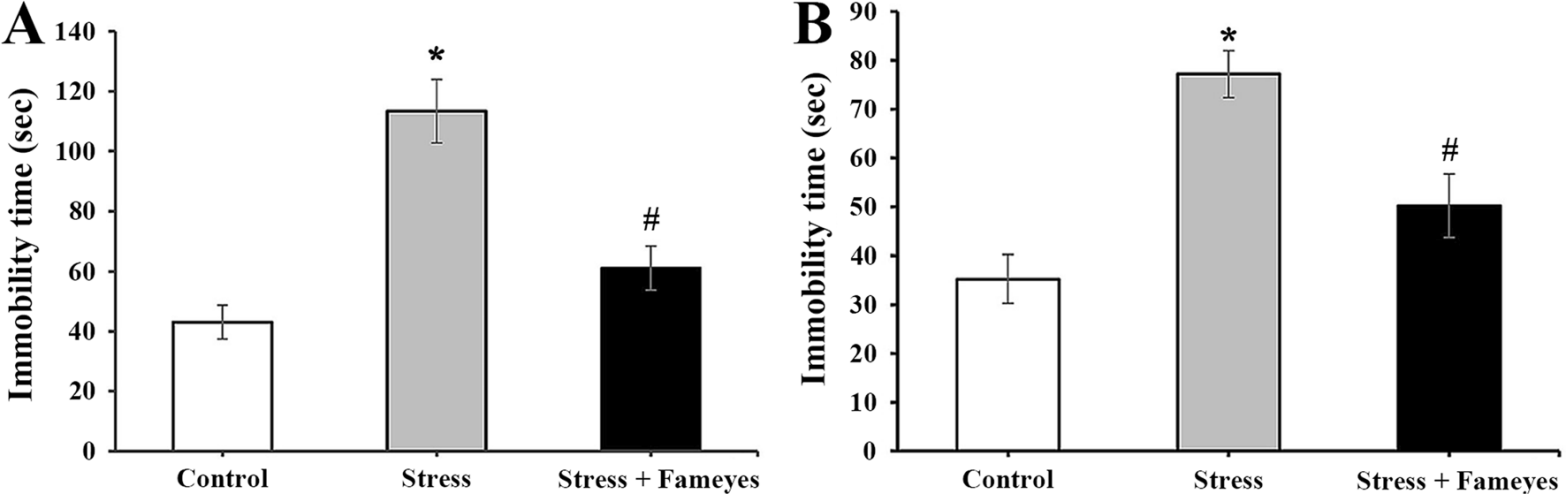
**A** Effects of Fameyes on immobility time of stressed rats in FST. The immobility time of the stress group is 113.4 ± 10.6 s, but it is considerably reduced to 61.1 ± 7.4 s in the stress + Fameyes group. Each bar represents the mean ± SEM (*n* = 10, respectively). *P < 0.05; compared with control group. ^#^*P* < 0.05; compared with stress group. **B** Effects of Fameyes on immobility time of stressed rats in TST. The immobility time of the stress group increases to 77.2 ± 4.8 s, but it is considerably reduced to 50.2 ± 6.5 s in the stress + Fameyes group. Each bar represents the mean ± SEM (*n* = 10, respectively). *P < 0.05; compared with control group. ^#^*P* < 0.05; compared with stress group

In this study, behavioral tests for stress in all groups were performed using forced swim test (FST) and tail suspension test (TST) (Fig. 4). In order to examine change in behavior when the rats are forced to swim, the immobility time of the rats was recorded using FST (Fig. 4A). The immobility time of the control group (neither subjected to stress nor Fameyes) was 43.1 ± 5.6 s. The immobility time of the stressed group was 113.4 ± 10.6 s; however, the immobility time was considerably reduced to 61.1 ± 7.4 s in the stress + Fameyes group. In order to examine changes in stress, depression and fatigue in despair, TST was conducted (Fig. 4B). The immobility time of the control group was 35.3 ± 5.0 s. The immobility time of the stress group increased to 77.2 ± 4.8 s, but was considerably reduced to 50.2 ± 6.5 s in the stress + Fameyes group.

### Effects of Fameyes on levels of ACTH, corticosterone, and serotonin in sera of stressed rats

**Fig. 5:**
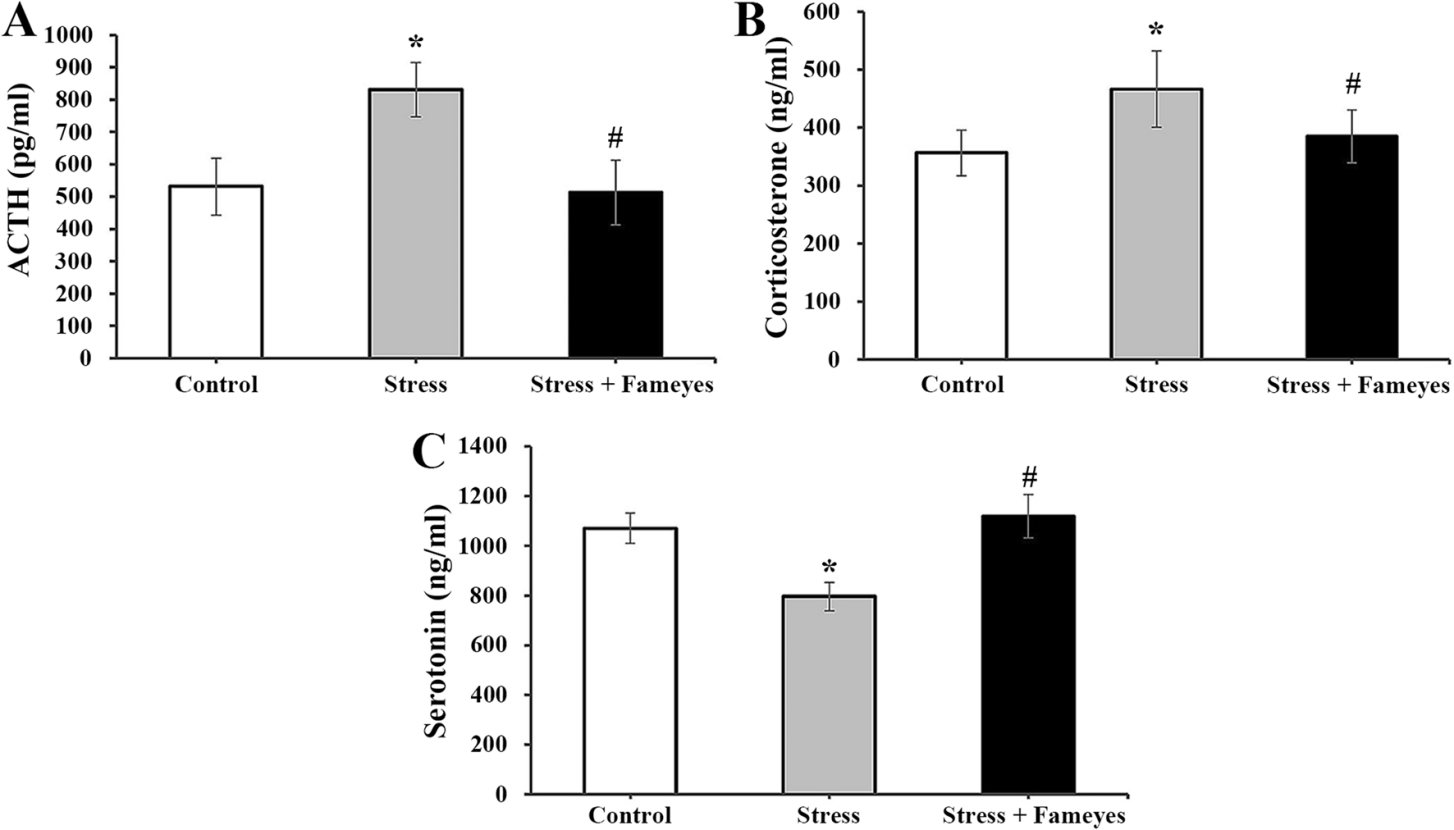
**A** Effects of Fameyes on serum level of ACTH in stressed rats. ACTH level in the stress group is 1.5 times that of the control group, but Fameyes treatment reducs to the level of the control group. Each bar represents the mean ± SEM (*n* = 10, respectively). *P < 0.05; compared with control group. ^#^*P* < 0.05; compared with stress group. **B** Effects of Fameyes on serum level of corticosterone in stressed rats. The corticosterone level of the stress group is 1.3 times that of the control group; however, the level in the stress + Fameyes group reduces to the level of the control group. Each bar represents the mean ± SEM (*n* = 10, respectively). *P < 0.05; compared with control group. ^#^*P* < 0.05; compared with stress group. **C** Effects of Fameyes on serotonin level in stressed rats. The serotonin level in the stress group was 0.7 times that of the control group, but Fameyes treatment increases the level to that of the control group. Each bar represents the mean ± SEM (*n* = 10, respectively). *P < 0.05; compared with control group. ^#^*P* < 0.05; compared with stress group

In order to examine the effects Fameyes, stress hormones were evaluated (Fig. 5). The serum concentration of ACTH, which promotes stress hormone secretion, was examined. As shown in Fig. 5A, ACTH level in the stress group was 1.5 times compared to that of the control group, but treatment with Fameyes resulted in a reduction of ACTH level, which was similar to that of the control group. As shown in Fig. 5B, the corticosterone (stress hormone) level of the stress group was 1.3 times compared to that of the control group. However, the corticosterone level of the stress + Fameyes group was similar to that of the control group. Change in serotonin, which supports physical and mental health, happiness and energy, was examined. As shown in Fig. 5C, the serotonin level of the stress group was 0.7 times compared to that of the control group, but the level in the stress + Fameyes group was similar to the control level.

## Discussion

In a previous study [15], Fameyes was observed to relieve mental stress in human subjects by improving test scores that measured the levels of depression, anxiety, well-being, and mental fitness. In addition to these subjective findings, the present study presents objective findings supporting the anti-stress activity of Fameyes through in vitro and in vivo experiments. Firstly, Fameyes inhibited apoptosis, and improved cell viability and the MMP of SH-SY5Y cells treated with corticosterone (400 μM) in a dose-dependent manner (Figs. 1, 2, 3). The effect was most pronounced when cells were treated with 100 μg/mL of Fameyes. Interestingly, this effect was observed only in the case of Fameyes treatment and only marginally when cells were treated with CMRE or EAPE individually (Fig. 1). Secondly, Fameyes substantially suppressed the harmful effects observed in the rats exposed to stressful conditions for four weeks. Fameyes decreased the immobility time in FST (Fig. 4A) and TST (Fig. 4B), decreased serum levels of ACTH (Fig. 5A) and corticosterone (Fig. 5B), and revived serotonin serum levels (Fig. 5C). Overall, these findings further support the anti-stress activity of Fameyes as observed in the previous human study.

Cortisol, a glucocorticoid, is a major stress hormone in humans. Cortisol is synthesized from corticosterone by 17 α-hydroxylase. However, in rodents, cortisol cannot be synthesized owing to the lack of this enzyme; thus, corticosterone acts as the stress hormone [6]. Therefore, in the present study, corticosterone was analyzed in cellular experiments because the rat model was used. Corticosterone was shown to damage cells by inducing apoptosis and decreasing cell viability and MMP. This cellular damage was prevented by Fameyes (Figs. 1, 2, 3).

The mechanism of the cell-damaging action of corticosterone is still unclear, but it may be due to the production of ROS. This assumption is based on the finding that EAPE, one of the components of Fameyes, suppressed ROS production in 3T3-L1 cells treated with methylisobutylxanthine, dexamethasone, and insulin [11]. Further, Fameyes was reported to lower serum levels of 8-hydroxyguanine in humans and inhibited brain damage induced by ischemia–reperfusion in gerbils [13, 14]. Both of these situations are typically associated with ROS production in the involved tissues. Therefore, the results of the in vitro experiments suggest that oxidative stress is a principal mechanism of the cell-damaging action of corticosterone, and Fameyes antagonizes this action by removing the generated ROS. This assumption is further supported by a recent report showing that YES‑10® predominantly contains antioxidants, such as scutellarin and chlorogenic acid [14].

The in vivo experiments showed that Fameyes inhibited the stress-induced stimulation of the hypothalamic–pituitary–adrenal (HPA) axis. HPA axis stimulation by stress was evidenced by increases in serum levels of ACTH (Fig. 5A) and corticosterone (Fig. 5B) and decrease in serum levels of serotonin (Fig. 5C) [2, 16, 17].

Questions about how Fameyes inhibits ACTH secretion induced by physical or mental stress and what its initial site of action is remain unanswered. The answers to these questions may be found in the antioxidant activity of Fameyes. It is well known that stress induces oxidative stress [18]. Moreover, the brain is one of the organs subjected to oxidative stress [19]. An oxidatively stressed brain is characterized by the expression and secretion of ACTH, secretion of corticosterone and decreased serotonin levels, which are factors that give rise to stress-related behavior [4, 5, 7].

## Conclusions

In conclusion, in vitro experiments showed anti-stress effects of Fameyes in cell viability, apoptosis, and mitochondrial membrane potential (MMP). In addition, in vivo experiments using rats showed anti-stress effects of Fameyes in blood and tissue levels of ACTH, corticosterone, and serotonin, as well as the immobility time in the forced swim and tail suspension tests. However, we did not specifically investigate which ingredient or ingredients showed anti-stress effects, although we reported that Fameyes contained chlorogenic acid and scutellarin major ingredients [14].

## Methods

### Preparation of Fameyes

CMRE and EAPE were prepared according to the patented protocols of Famenity Co., Ltd. The procedure of Fameyes was described in the published papers [13, 14]. In brief, both plants had been cultivated in Metro-Mix potting soil with a slow releasing fertilizer (Osmocote Plus®, PlantersPlace, Bloomington, IN, USA) for six weeks. Their leaves were respectively harvested and washed with pure water. Next, the leaves were dried at 50℃ and powderized (< 1 mm) using a grinder (M20, IKA, Staufen, Germany). The leaves (150 g, respectively) were extracted with seven-fold volume of 50% ethyl alcohol for one hour and refluxed two times (2 h/refulx). The extract was filtrated and concentrated to be fine powder using a rotary evaporator and stored at 4˚C. The extract was finally mixed with 1:1 of ratio to be Fameyes.

### Cell culture and experimental groups

SH-SY5Y cells, which are human neurofibroma cell line, provided by Korea Cell Bank (Seoul, South Korea; Catalogue No., 22266) were cultured in a Dulbecco's Modified Eagle's Medium (DMEM; Biowest, Nuaillé, France) containing 10% fetal bovine serum (FBS; Biowest) and 1% penicillin and streptomycin (Thermo Fisher Scientific, Waltham, MA, USA) at 37℃ and was supplied with 5% CO_2_ until the cell density reached 90% saturation. These cells were divided into five groups: (1) control group, (2) cells treated with corticosterone, (3) cells treated with CMRE and corticosterone, (4) cells treated with EAPE and corticosterone, and (5) cells treated with Fameyes and corticosterone. The cells were treated with 25, 50, or 100 μg/ml of CMRE, EAPE, or Fameyes for one hour and then treated with 400 μM corticosterone for 24 h. These cells were used for cell viability and MMP assays, as well as Hoechst 33342 staining to observe chromatin condensation and splitting.

### Cell viability test

SH-SY5Y cells were seeded in a 96-well plate (Nunc A/S, Roskilde, Denmark) at a density of 2.5 × 10^4^ cells/well and cultured for 24 h. After changing the culture medium, the cells were treated as described in the previous section, and cell viability assay was performed using Cell Counting Kit 8 (Dojindo Molecular Technologies, Rockville, MD, USA) according to the manufacturer’s protocol (The cells were treated with CCK-8 cell viability assay solution in volumes corresponding to 10% of the treated culture medium and cultured for additional two hours). Light absorbance at 450 nm of each well was measured using an Infinite M200 PRO Nano Quant microplate reader (TECAN, Zurich, Switzerland), and cell viability was presented as ratios relative to the control group (%).

### Hoechst 33342 staining

In order to observe the pattern of apoptosis induced by corticosterone-induced stress, Hoechst 33342 staining was used to stain nuclei and observe chromatin condensation and splitting. SH-SY5Y cells were seeded in 35-mm dishes at a density of 5 × 10^5^ cells/dish and cultured for 24 h. After changing the culture medium, the cells were treated as described in the previous section and stained with Hoechst 33342 (Sigma-Aldrich, St. Louis, MO, USA) by adding 5 μg/mL of the dye and incubating for five minutes at 37℃ in the dark. Morphological changes in cell nuclei were observed using the EVOS FL Imaging System (Life Technologies, Carlsbad, CA, USA).

### Assessment of MMP (∆Ψm)

In order to investigate changes in mitochondrial membrane potential (MMP, ∆Ψm), tetramethylrhodamine ethyl ester (TMRE, sigma) was used according to the manufacturer's protocol. SH-SY5Y cells were seeded in a 96-well black plate at a density of 2.5 × 10^4^ cells/well and cultured for 24 h. After changing the culture medium, the cells were treated as described in the previous section, and the MMP assay was performed using tetramethylrhodamine ethyl ester (TMRE; Sigma-Aldrich, MO, USA) according to the manufacturer’s protocol. Briefly, TMRE (200 nM) was added to the cells, which were then incubated for 15 min and washed with phosphate buffered Saline (PBS, pH 7.4). Fluorescence was measured using a fluorescence microplate reader (Glomax, Promega, Madison, WI, USA).

### Experimental animals

Specific pathogen-free (SPF) male Sprague–Dawley (SD) rats aged seven weeks (180 ± 20 g) were provided by Samtako Bio Korea (Osan, Korea). The rats were housed in a facility maintained at 23 ± 2℃ and 50 ± 10% relative humidity under 12-h light/dark cycles. Solid feed (Samtako Bio Korea) and drinking water were provided ad libitum. The rats were acclimatized for one week prior to the experiments. The care of the rats and all experimental procedures were approved by the Institutional Animal Care and Use Committee of Chung-Ang University (Approval No. 2017-00014). Humane endpoints were not established as the protocol of the current animal experiment did not cause any severe pain or stress.

### Induction of stresses in rats

The rats were randomly divided into three groups (*n* = 10, respectively): (1) control group, (2) stress group, and (3) stress + Fameyes group. The rats in the control group were neither subjected to any kind of stress nor administered Fameyes. The rats in the stress group were subjected to seven kinds of stresses for four weeks as follows: fasting for 24 h every other day, water supply cut off for 24 h every other day, cage tilted by 45º for seven hours every other day, overnight lighting every other day, wet sawdust bedding for 24 h every other day, activity restriction for two hours every other day, and exposure to foreign objects like plastic pieces for 24 h every other day. Using a curved feeding needle (16 gauge; 100 mm of length; Fine Science Tools, Inc. Foster City, CA, USA), the rats in the stress + Fameyes group were orally administered 50 mg/kg of Fameyes dissolved in water once a day for four weeks of the stress period. The rats in each group were tested for their ability to tolerate forced swimming and tail suspension. At four weeks after stress induction and Fameyes treatment, the rats were deeply anesthetized using pentobarbital (150 mg/kg, intraperitoneal injection) and blood (3 mL) was taken from the abdominal aorta. After blood collection, death of the rats was verified by cessation of breath. The colledted blood was centrifuged within one hour at 3000 rpm for 10 min to obtain serum.

### FST and tail TST

These tests were used to measure the ability of the rats to tolerate harsh conditions, such as forced swimming and tail suspension, by measuring the time of immobility. FST was conducted as per the protocol designed by Pege et al. [20]. A clear acrylic cylinder (diameter = 25 cm, height = 50 cm) was filled with water at 23℃ up to 30 cm. The rats were then placed in the cylinder for 15 min. A day later, the rats were placed in the cylinder again for five minutes, during which the immobility time of each rat was measured. In the TST [21], the rats were hung upside down by their tails for six minutes. No measurement was made in the first two minutes of tail suspension, and the immobility time was measured during the remaining four minutes.

### Analysis of ACTH, corticosterone, and serotonin in sera

The rats exposed to various stressors for four weeks were anesthetized with a mixture of N_2_O, O_2_ and isoflurane. The abdomen was then incised along the midline, and blood was collected from the abdominal aorta. The blood samples were centrifuged for 10 min at 3000 rpm within one hour to separate the plasma. The separated plasma was then stored at -80℃ until analysis. Corticosterone concentrations were measured using a corticosterone enzyme immunoassay kit (Immunodiagnostic Systems, Boldon, UK). ACTH levels were quantified by radioimmunoassay using an ACTH kit (BRAHMS, Hennigsdorf, Germany) and γ-counter (Cobra Quantum E5010; Packard BioScience Company, Meriden, Connecticut, USA). Serotonin was analyzed by HPLC (Alliance; Waters Corporation, Milford, MA, USA) using a serotonin kit (RECIPE, Munich, Germany).

### Statistical analysis

All results are presented as the mean ± standard deviation. Analysis of variance was used to test for differences between groups, and Tukey’s test was used for post-hoc analysis. Differences were considered statistically significant at *P* < 0.05.

## Data Availability

The data presented in this study are available on request from the corresponding authors.

## Abbreviations

ACTH: Adrenocorticotropic hormone
CMR: *Clematis mandshurica* Rupr
CMRE: *Clematis mandshurica* Rupr. Extract
EAP: *Erigeron annuus* (L.) Pers.
EAPE: *Erigeron annuus* (L.) Pers. Extract
ER: Endoplasmic reticulum
FBS: Fetal bovine serum
FST: Forced swim test
HPA: Hypothalamic–pituitary–adrenal
MMP: Mitochondrial membrane potential
ROS: Reactive oxygen species
SD: Sprague–Dawley
SEM: Standard error mean
SPF: Secific pathogen-free
TBS: Tris-buffered saline
TMRE: Tetramethylrhodamine ethyl ester
TST: Tail suspension test
